# 

**Published:** 1917-12

**Authors:** 


### TABLE OF CONTENTS

Crawford Williamson Long and Ether (Cont'd). ---------- 253
The Training of the General Practitioner for Obstetrics --- 261
American Proctologist Society ---------------------------- 268
The Place of the Proctologist in a Diagnostic Group ------ 269
Adult Rectal Prolapse; Two Cases and a Contrast ---------- 271
Relation of Hemorrhoidal Disease to the Health Balance -- 272
The Principals Underlying the Clamp and Operation for Hemorrhoids ---------------------------------- 273
Original Research Work on Pruritus Ani ------------------- 273
Adenomyoma of the Rectum --------------------------------- 275
Pellagra, the Pellagrous Intestine and Pericolic Veins ---- 277
Non-proctology. A Glimpse into the Future ---------------- 278
Report of a Case of Idiosyncracy to Quinine and Urea Hydrochloride ------------------------------------ 278
The Non-Surgical Treatment of Splanchnoptosis ------------ 279
Neglected Rectal Examination ----------------------------- 280
Trade Commision Acts on Salvarsan Patent ----------------- 281
War Prices Depriving Babies of Milk ---------------------- 282
Medical Items -------------------------------------------- 284

